# Supplementary material for: Novel Soloxolone Amides as Potent Anti-Glioblastoma Candidates: Design, Synthesis, In Silico Analysis and Biological Activities In Vitro and In Vivo
Source: Pharmaceuticals (Basel). 2022 May 14;15(5):603. doi: 10.3390/ph15050603 (PMC9145754; doi:10.3390/ph15050603)
Supplement: Supplementary file 1 [file pharmaceuticals-15-00603-s001.zip › pharmaceuticals-1714641-supplementary.pdf]

Article

Novel Soloxolone Amides as Potent Anti-Glioblastoma Candidates: Design, Synthesis, In Silico Analysis and Biological Activities In Vitro and In Vivo

Andrey V. Markov [1,*], Anna A. Ilyina [1,2], Oksana V. Salomatina [1,3], Aleksandra V. Sen'kova [1], Alina A. Okhina [2,3], Artem D. Rogachev [2,3], Nariman F. Salakhutdinov [3] and Marina A. Zenkova [1]

[1] Institute of Chemical Biology and Fundamental Medicine, Siberian Branch of the Russian Academy of Sciences, 630090 Novosibirsk, Russia; a.ilina8@g.nsu.ru (A.A.I.); ana@nioch.nsc.ru (O.V.S.); senkova_av@niboch.nsc.ru (A.V.S.); marzen@niboch.nsc.ru (M.A.Z.)

[2] Faculty of Natural Sciences, Novosibirsk State University, 630090 Novosibirsk, Russia; aokhina@nioch.nsc.ru (A.A.O.); rogachev@nioch.nsc.ru (A.D.R.)

[3] N.N. Vorozhtsov Novosibirsk Institute of Organic Chemistry, Siberian Branch of the Russian Academy of Sciences, 630090 Novosibirsk, Russia; anvar@nioch.nsc.ru

* Correspondence: andmrkv@gmail.com; Tel.: +7-383-363-51-61

Supplementary File 1. NMR Spectrum of synthesized compounds

Spectrum of Soloxolone **1,** ^{1}H NMR, 500MHz, $CDCl_3$

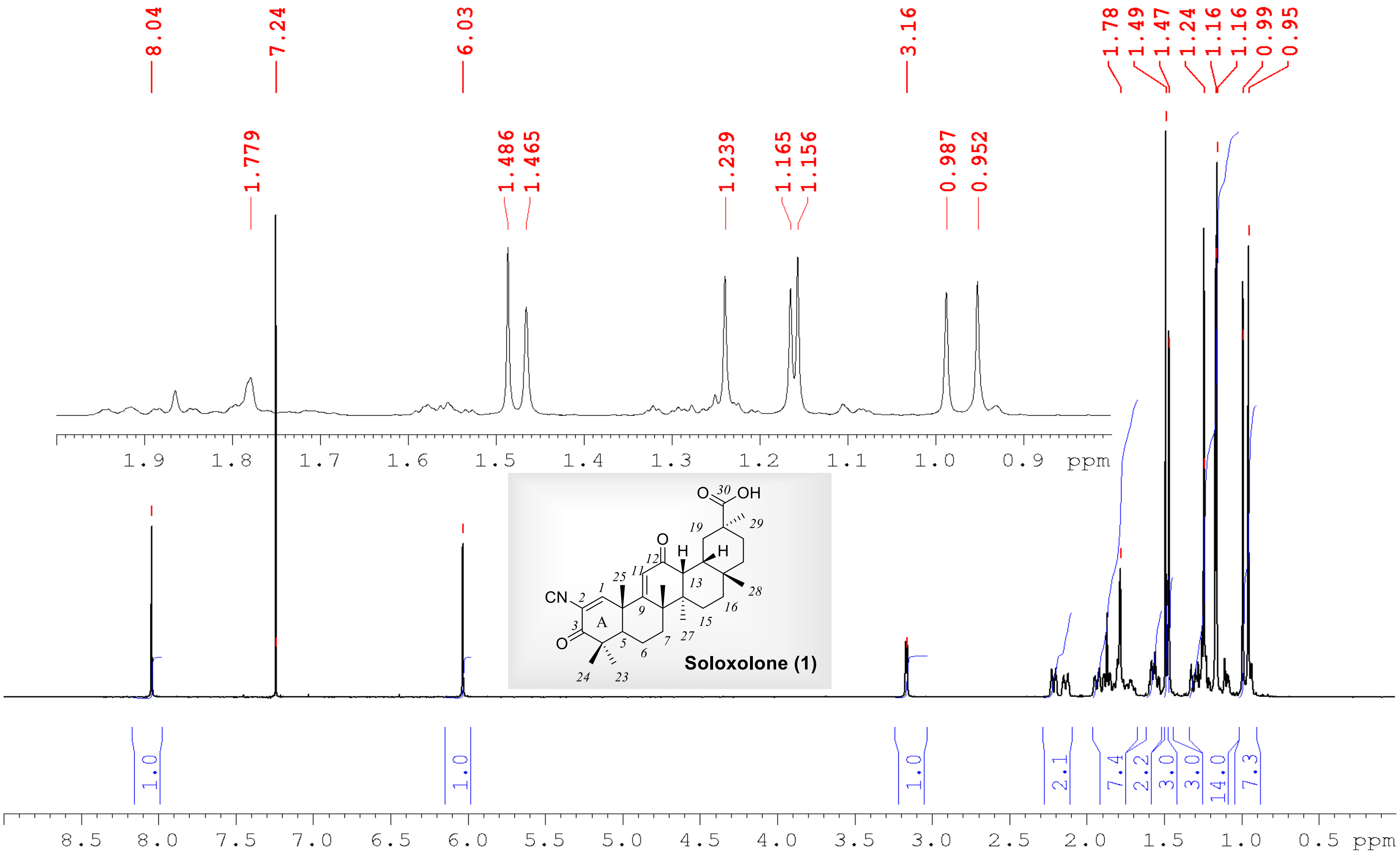

Spectrum of Soloxolone **1,** ^{13}C NMR, 125MHz, $CDCl_3$

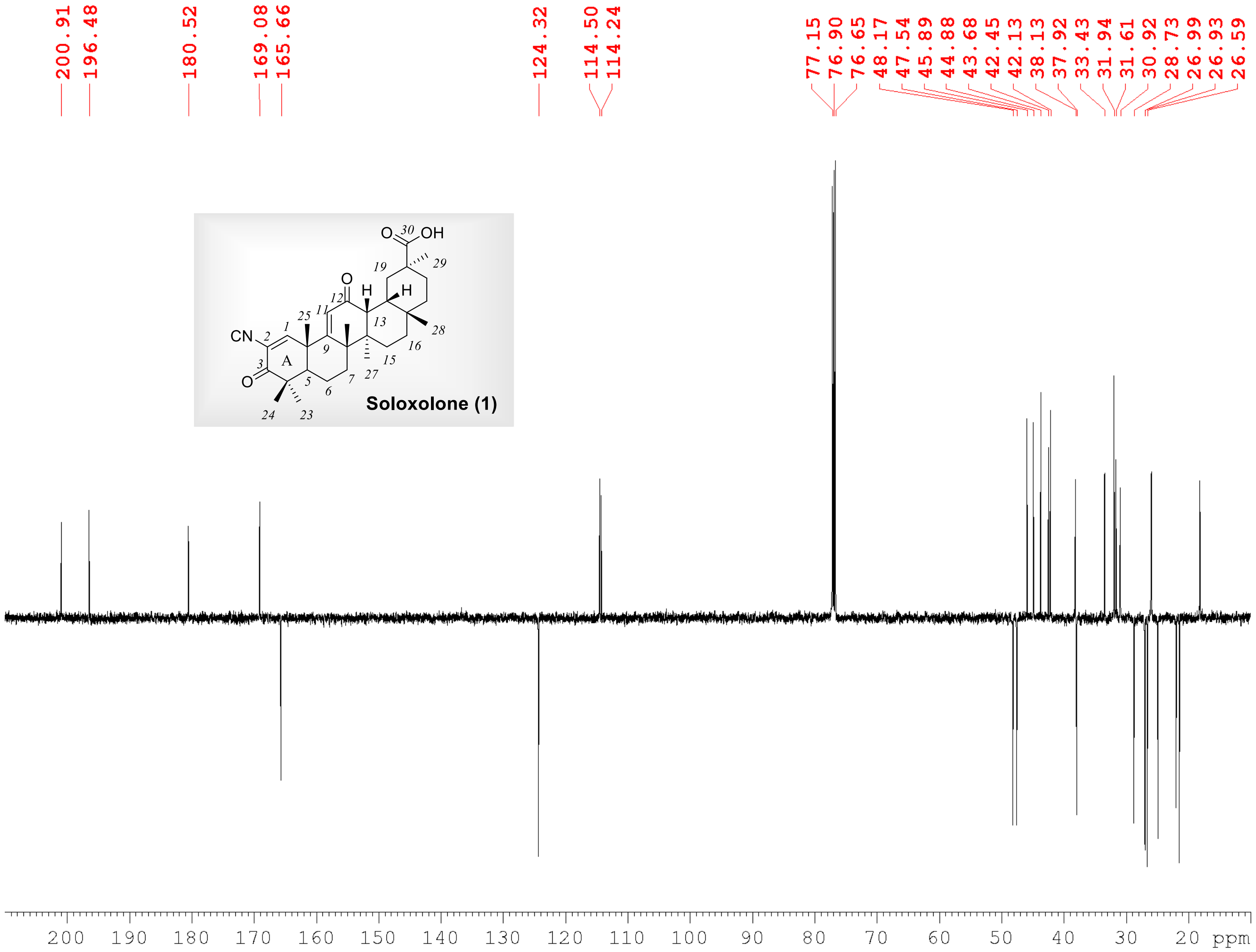

Spectrum of Compound **2**, ^{1}H NMR, 500MHz, $CDCl_3$

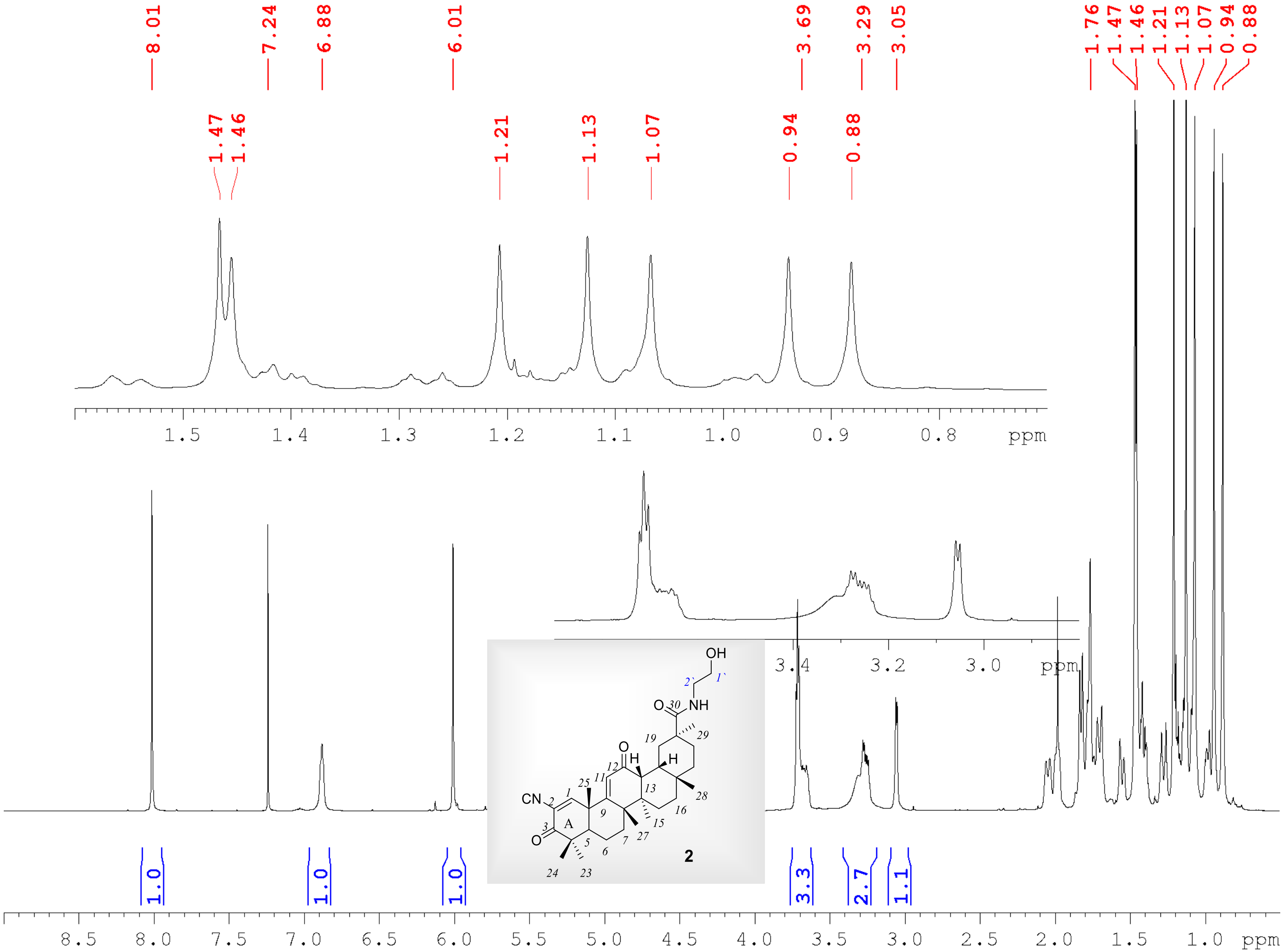

Spectrum of Compound **2,** ^{13}C NMR, JMOD, 125MHz, $CDCl_3$

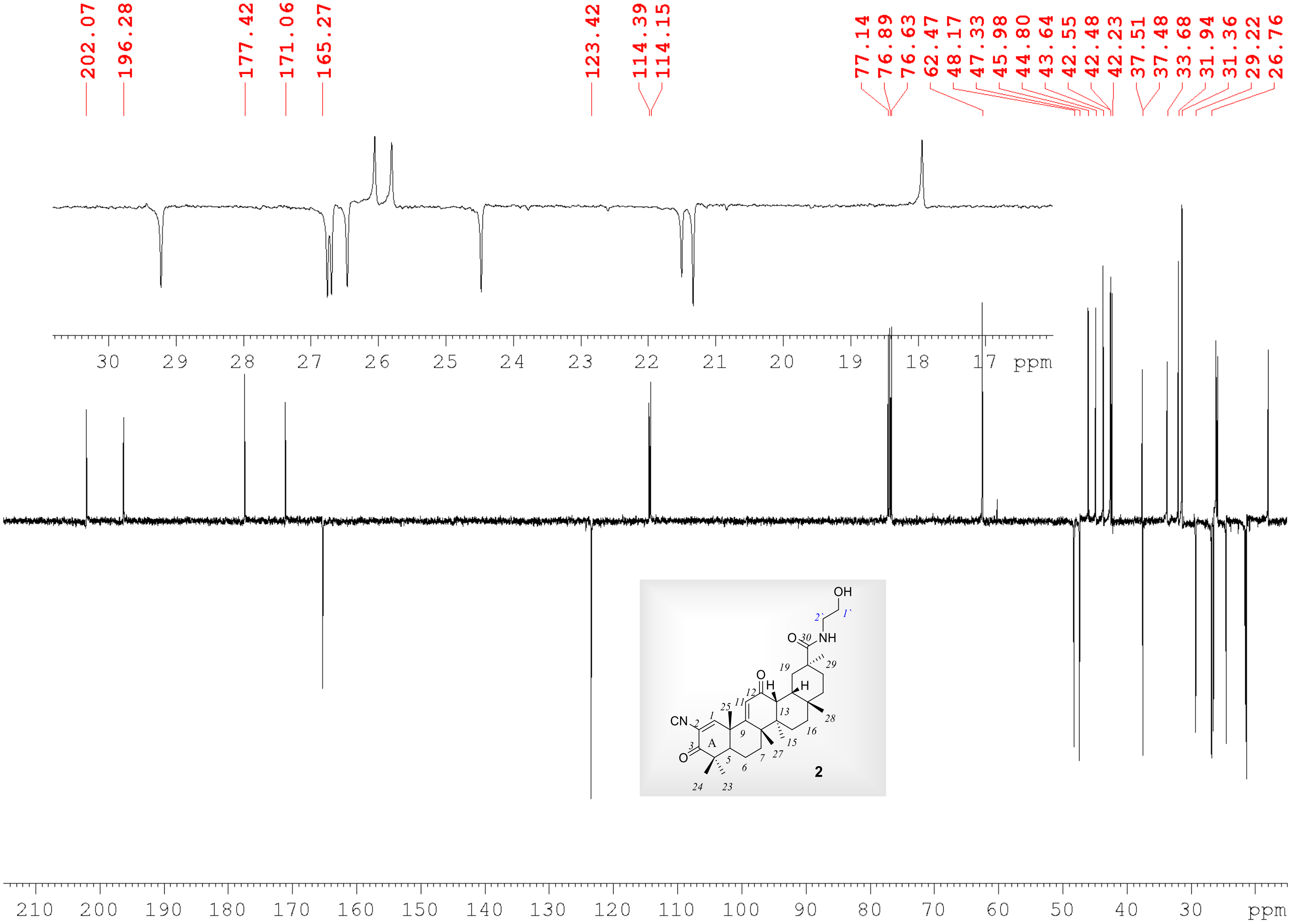

Spectrum of Compound **3,** ^{1}H NMR, 500MHz, $CDCl_3$

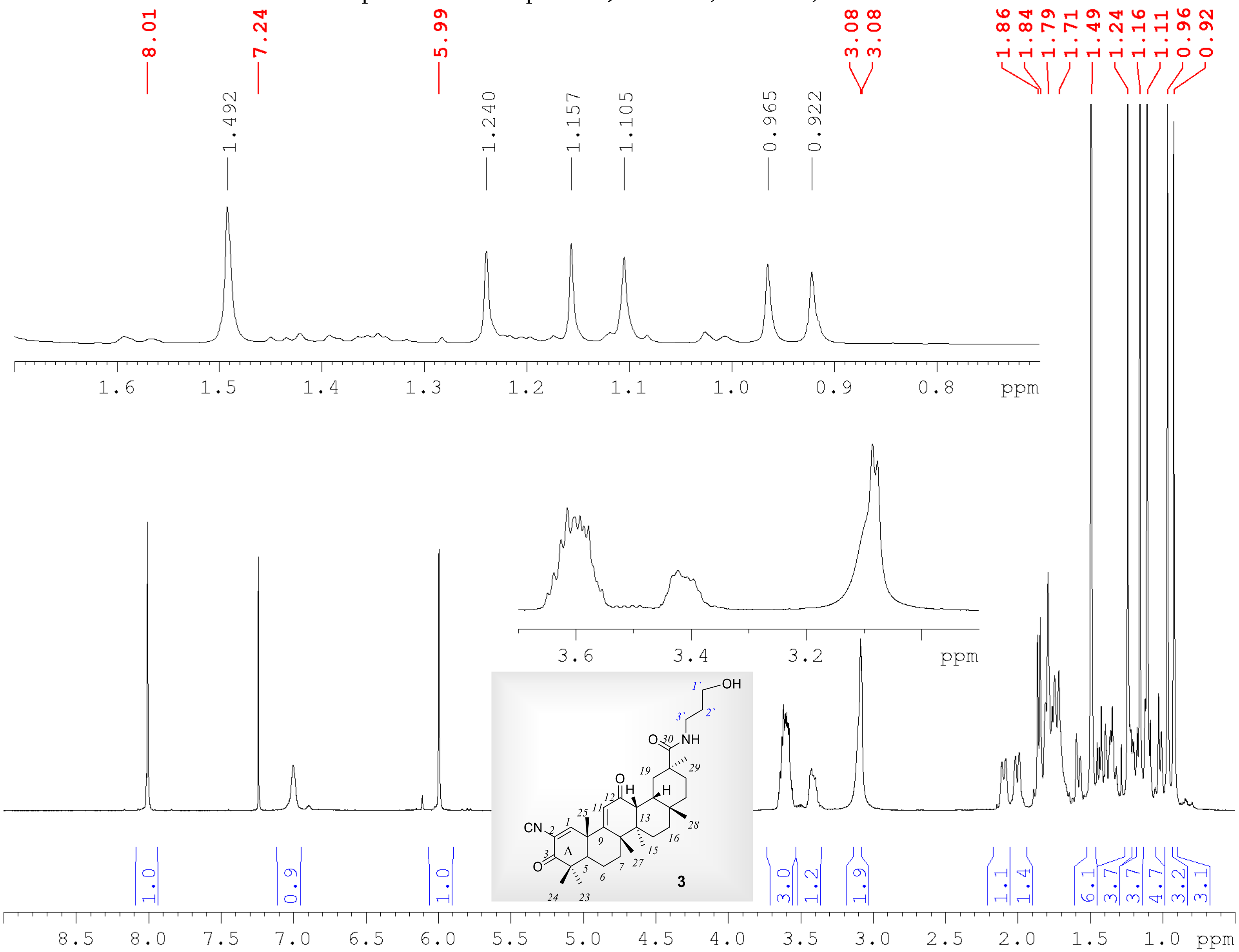

Spectrum of Compound **3**, ^{13}C NMR, JMOD, 125MHz, $CDCl_3$

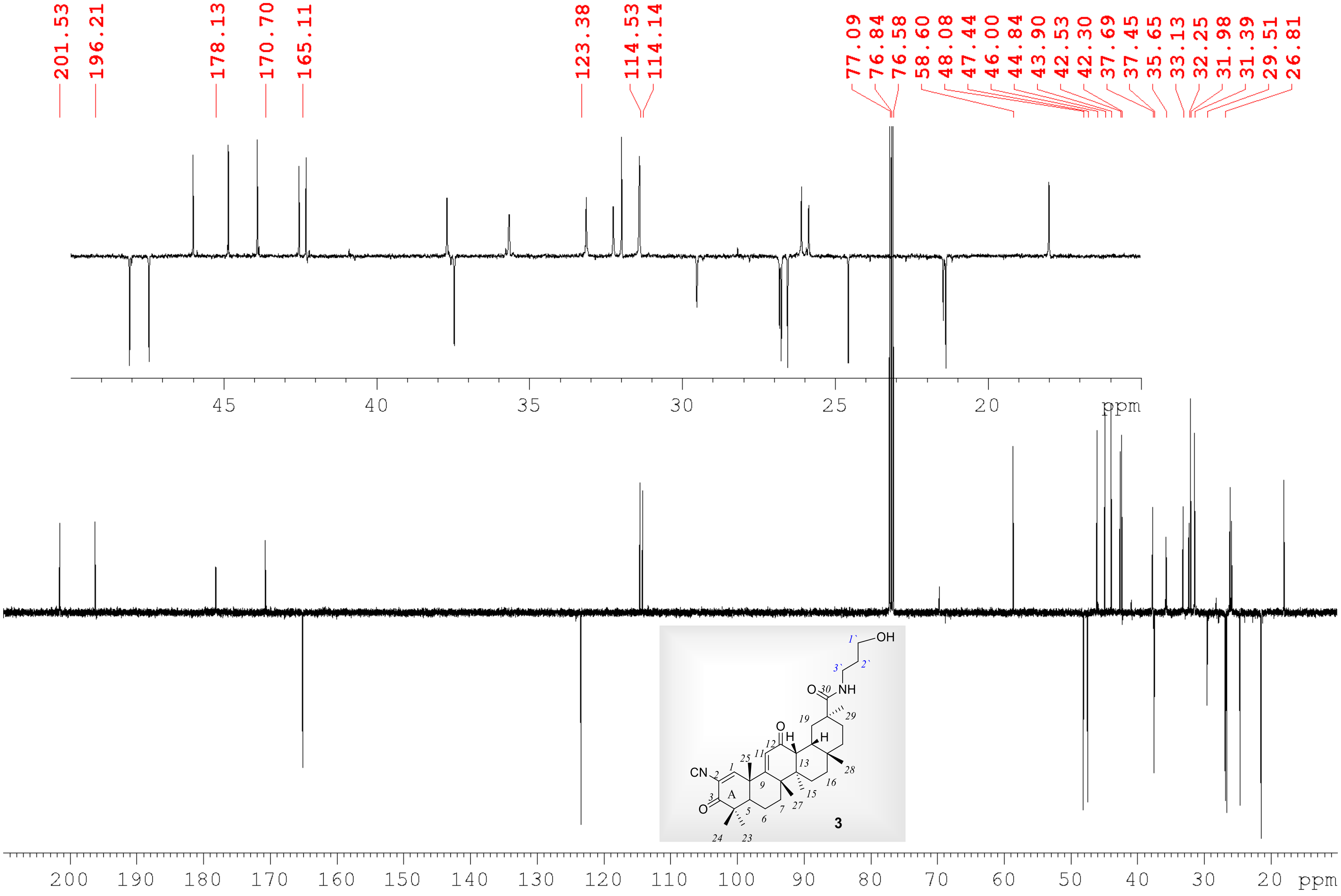

Spectrum of Compound **4**, ^{1}H NMR, 500MHz, $CDCl_3$

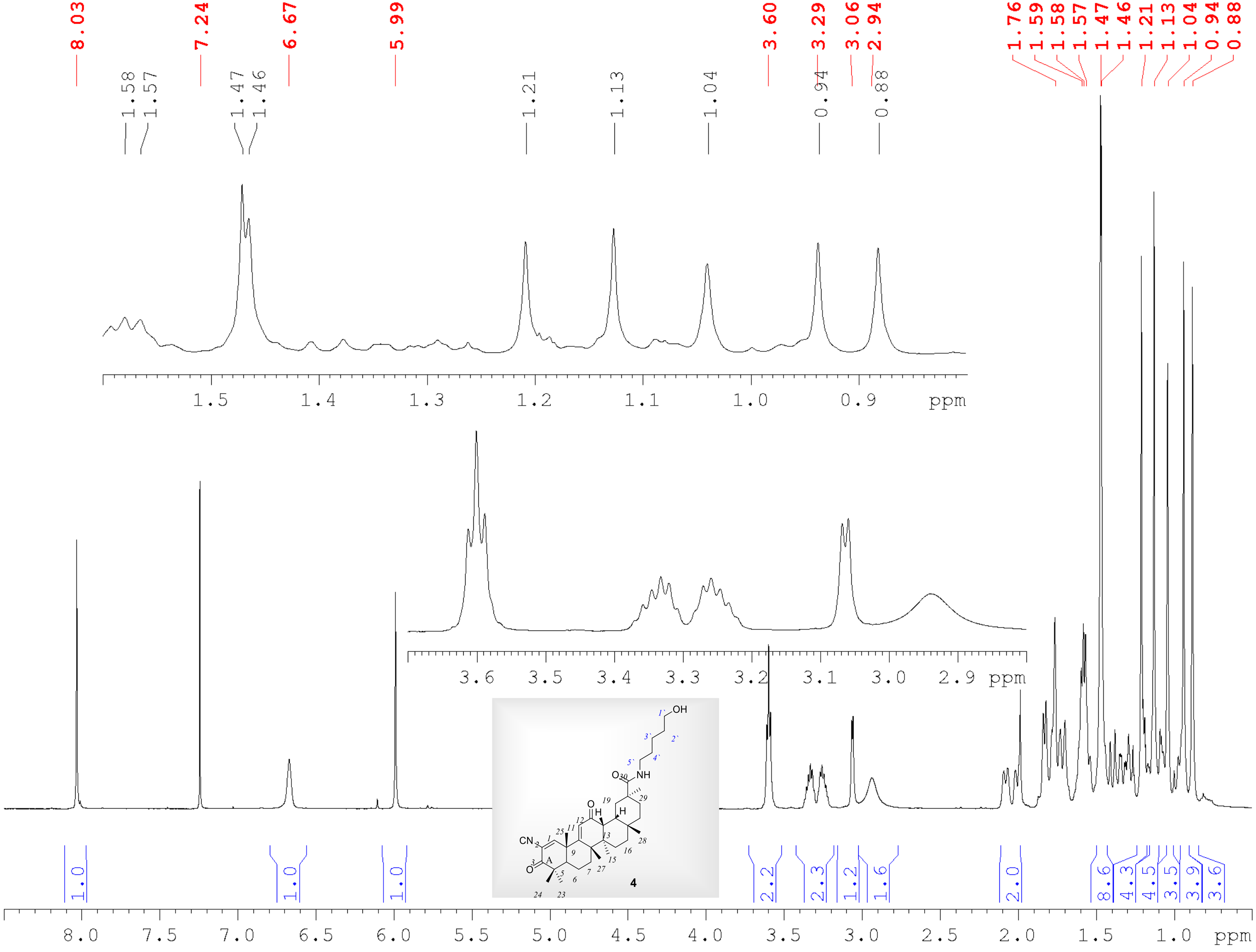

Spectrum of Compound **4**, ^{13}C NMR, JMOD, 125MHz, $CDCl_3$

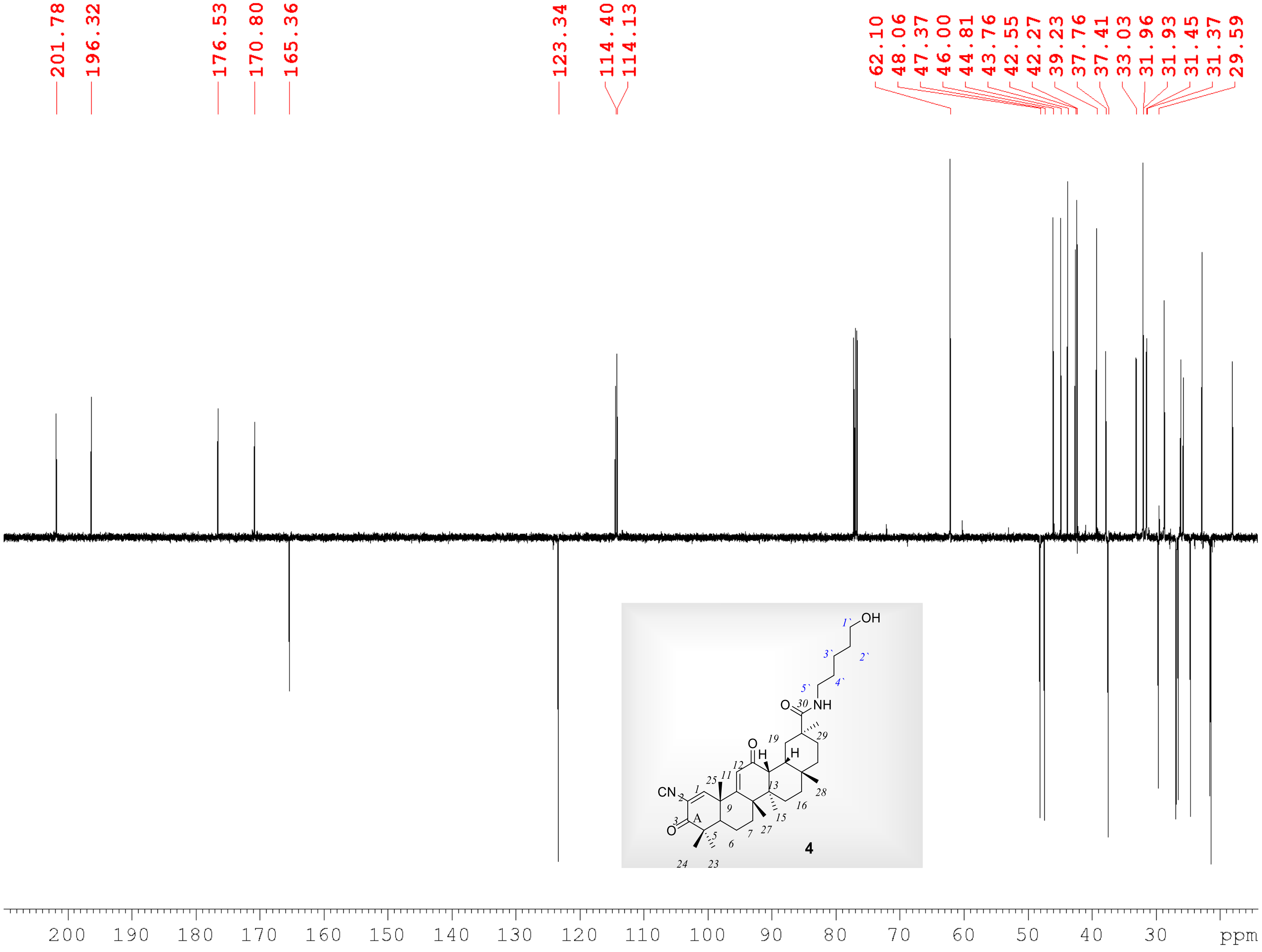

Spectrum of Compound **5,** ^{1}H NMR, 400MHz, $CDCl_3$

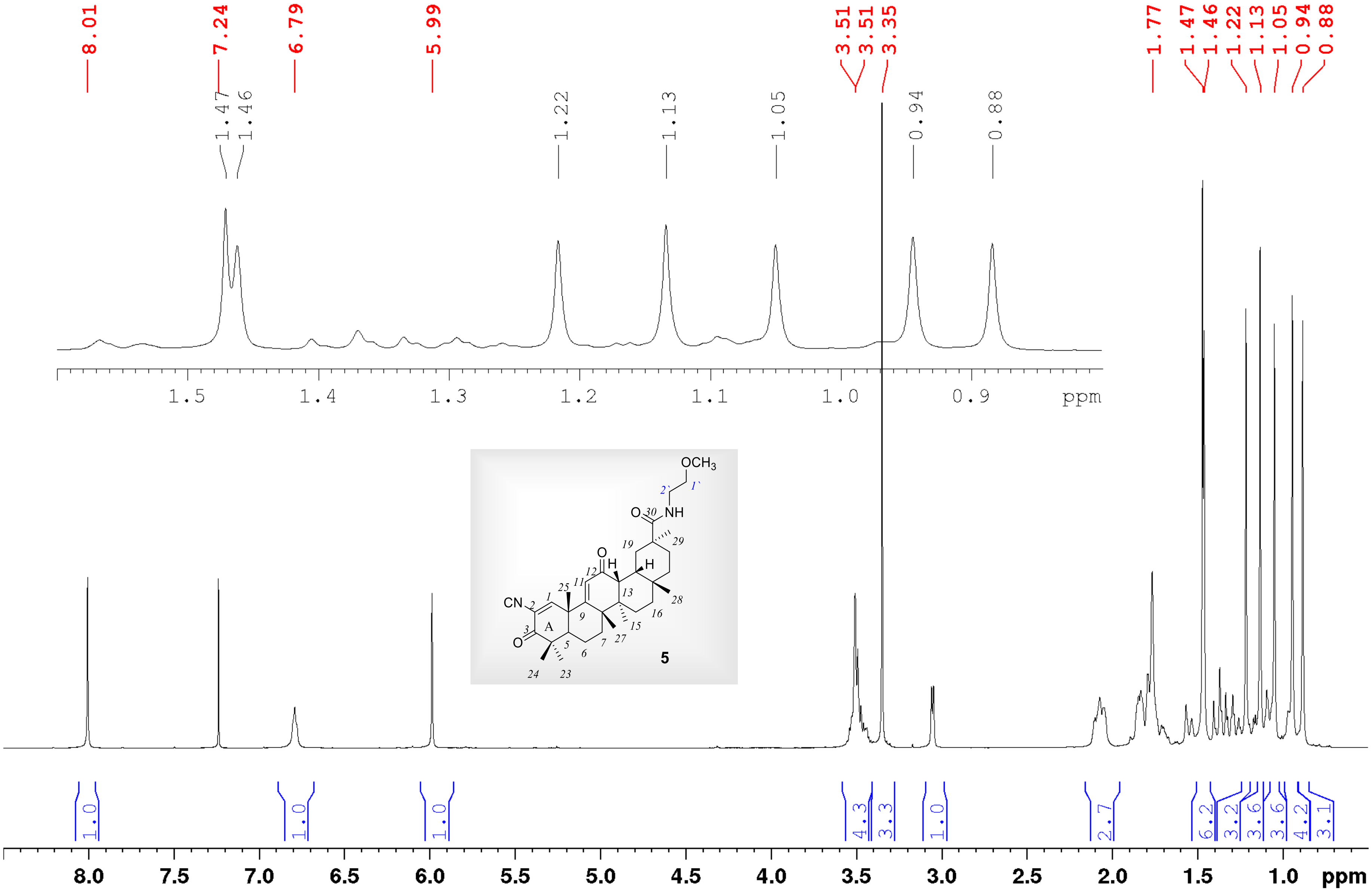

Spectrum of Compound **5,** ^{13}C NMR, JMOD, 100MHz, $CDCl_3$

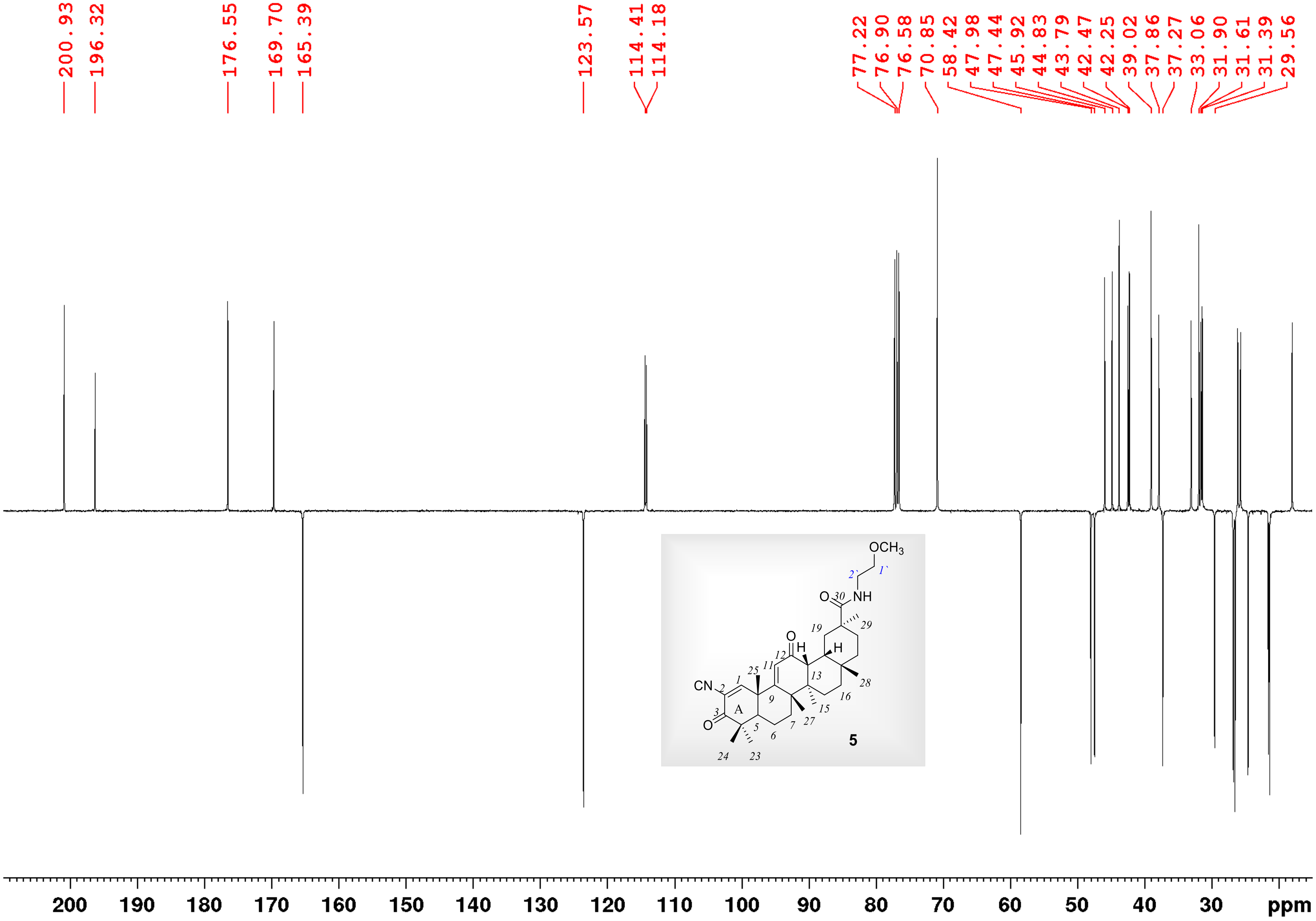

Spectrum of Compound **6,** ^{1}H NMR, 400MHz, CDCl$_3$

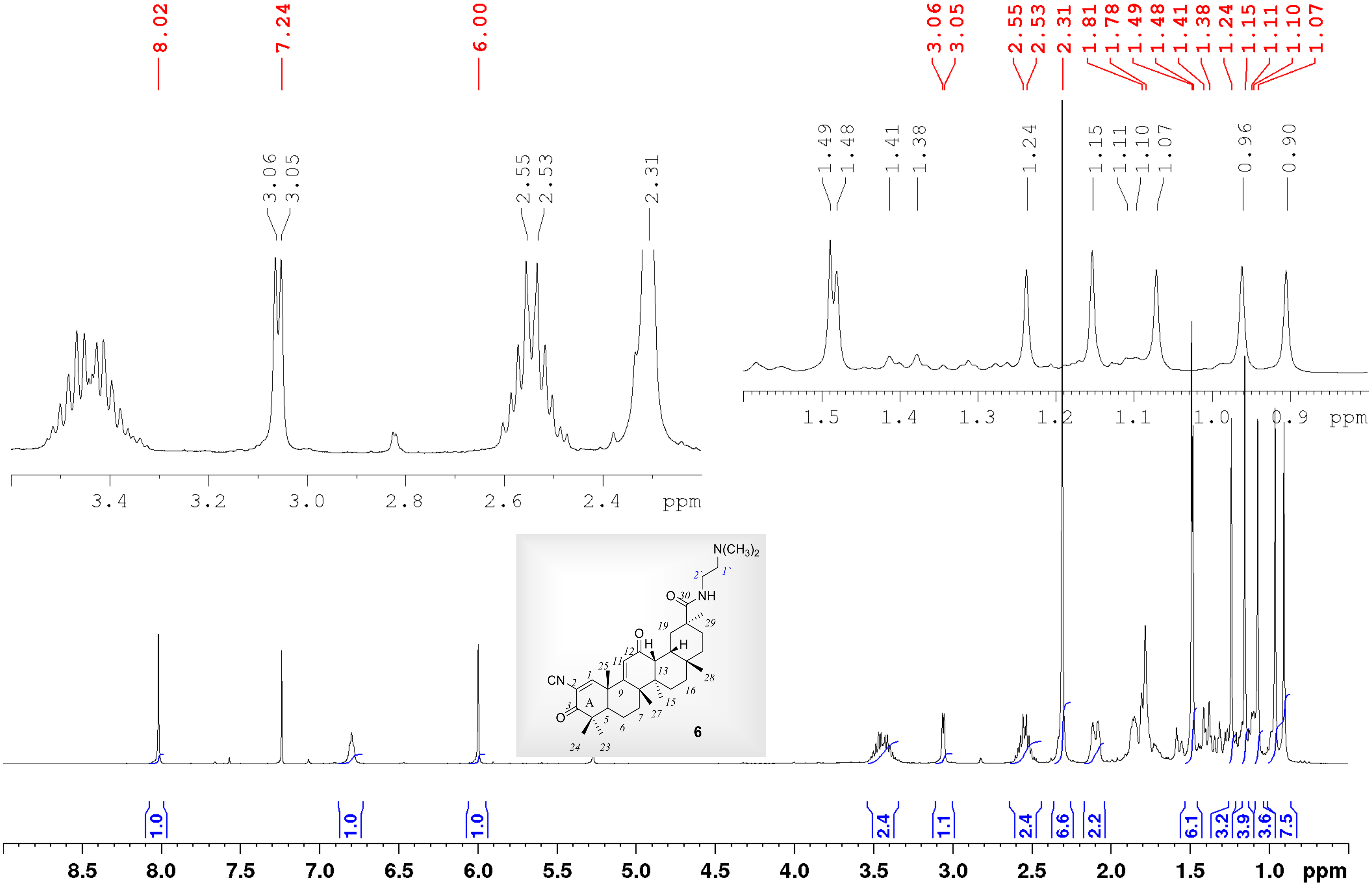

Spectrum of Compound **7,** ^{1}H NMR, 400MHz, $CDCl_3$

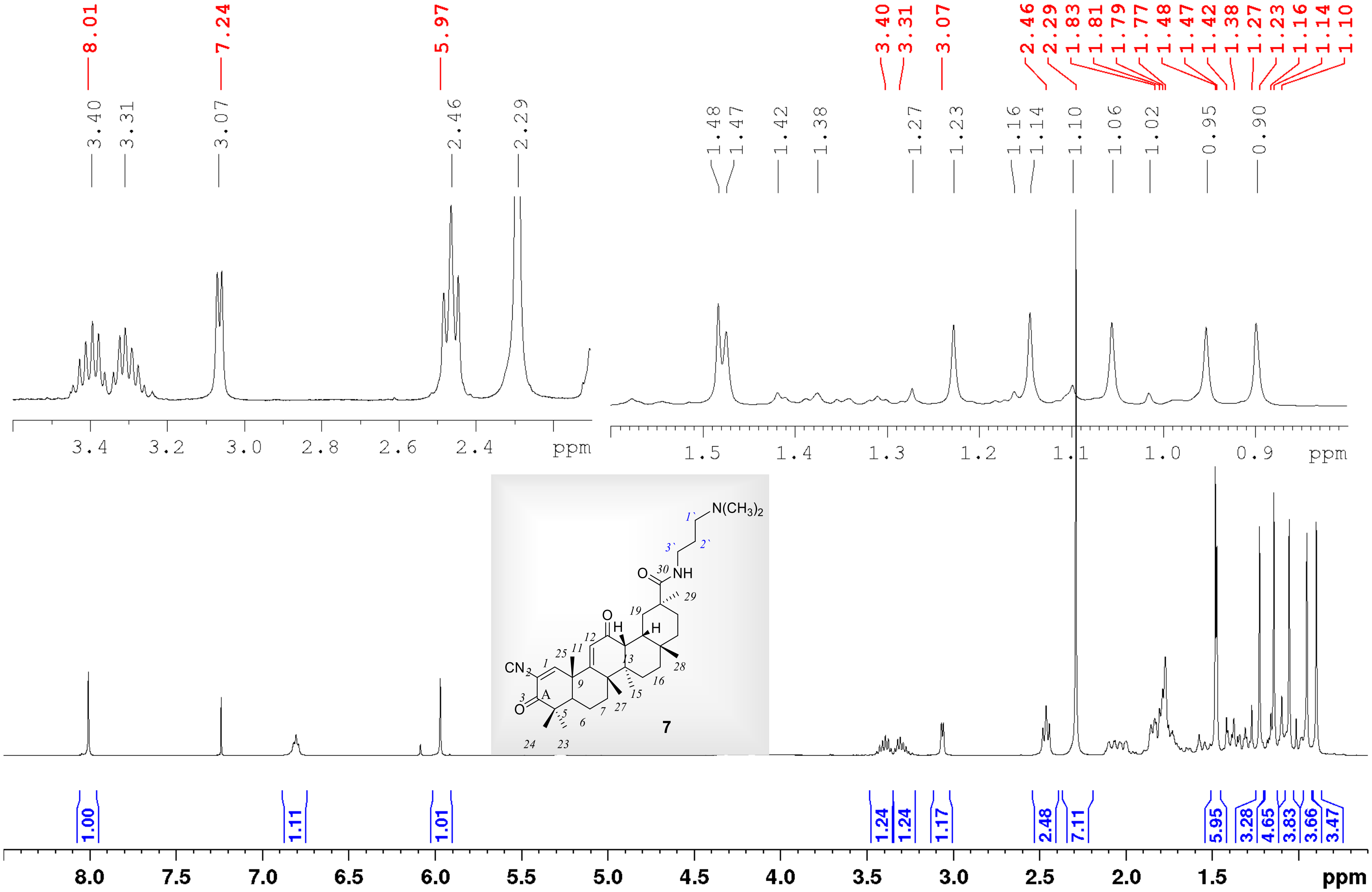

Spectrum of Compound **7,** ^{13}C NMR, JMOD, 400MHz, $CDCl_3$

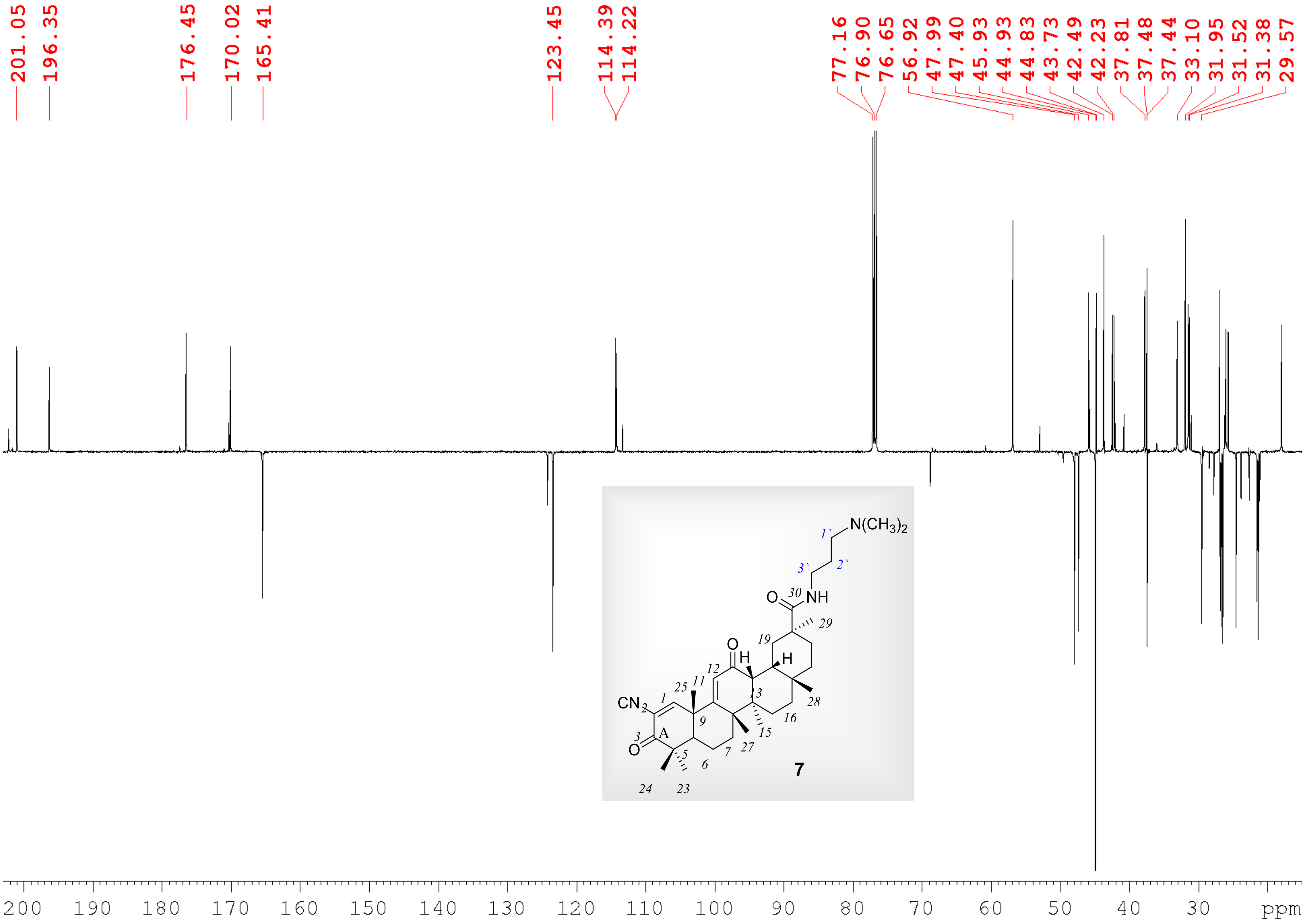

Spectrum of Compound **8,** ^{1}H NMR, 300MHz, $CDCl_3$

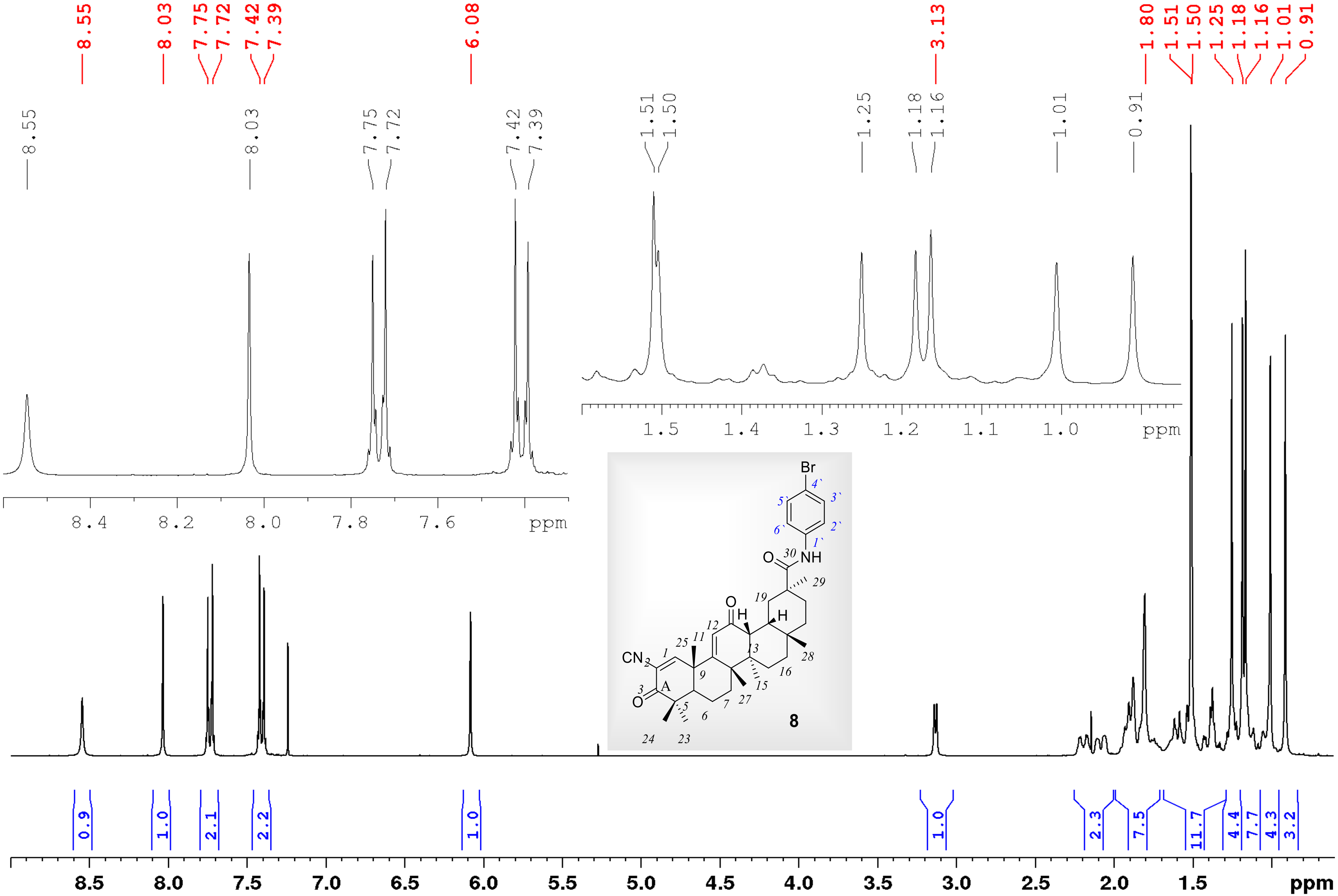

Spectrum of Compound **8,** ^{13}C NMR, JMOD, 300MHz, $CDCl_3$

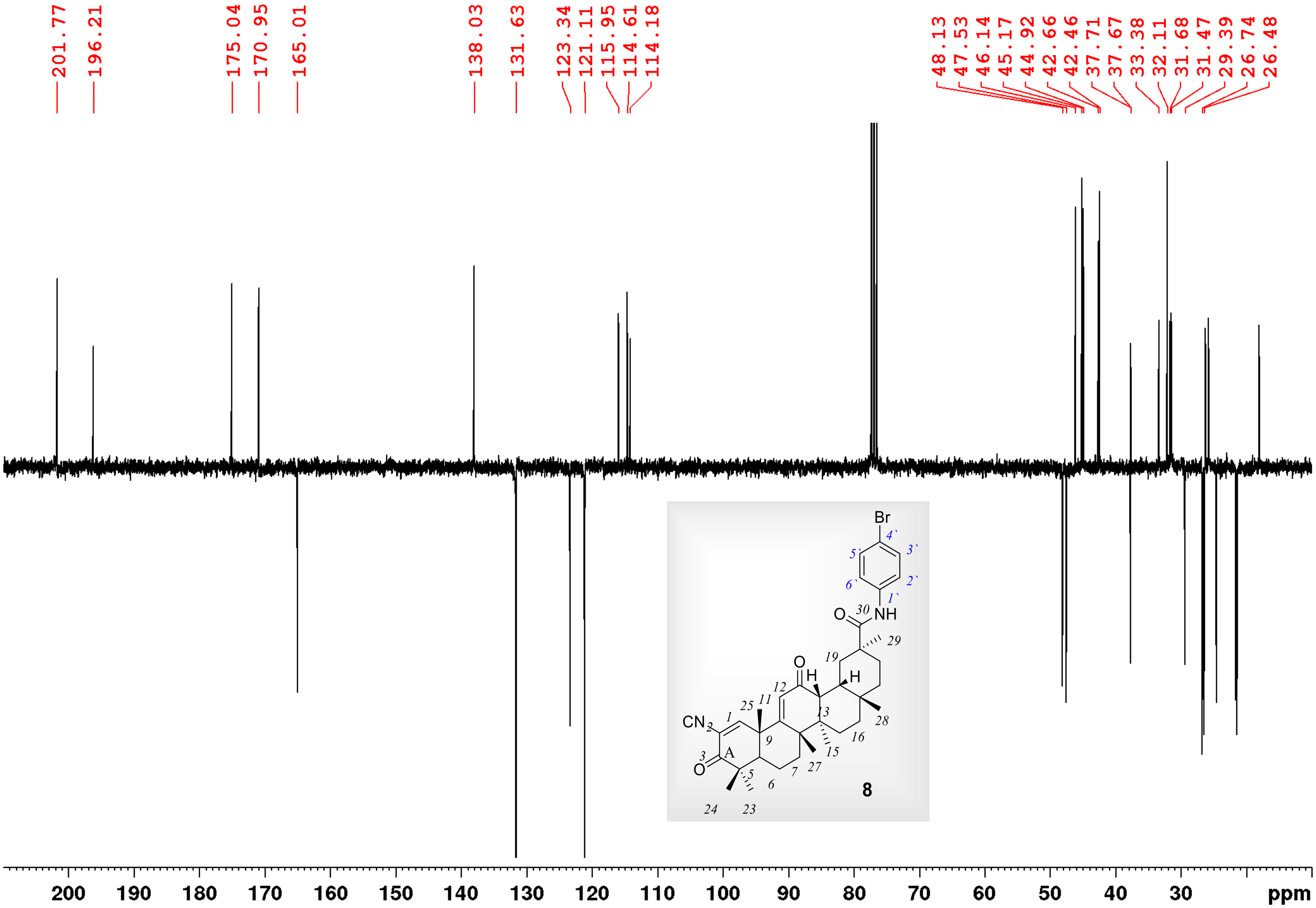
201.77
196.21
175.04
170.95
165.01
138.03
131.63
123.34
121.11
115.95
114.61
114.18
48.13
47.53
46.14
45.17
44.92
42.66
42.46
37.71
37.67
33.38
32.11
31.68
31.47
29.39
26.74
26.48
200
190
180
170
160
150
140
130
120
110
100
90
80
70
60
50
40
30
ppm
Br
NH
O
CN
A
8

Spectrum of Compound **9**, ^{1}H NMR, 400MHz, $CDCl_3$

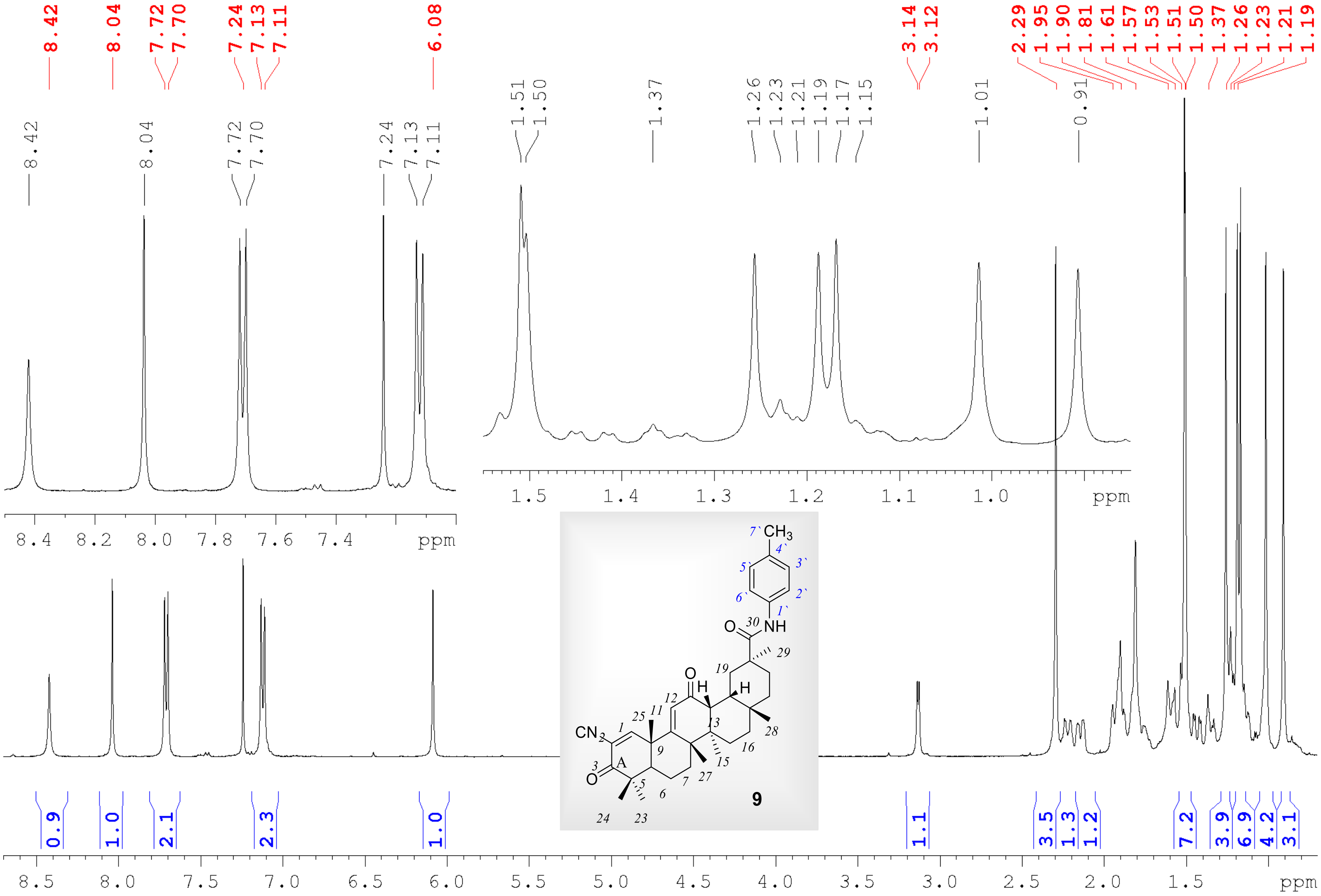

Spectrum of Compound **9,** ^{13}C NMR, JMOD, 100MHz, $CDCl_3$

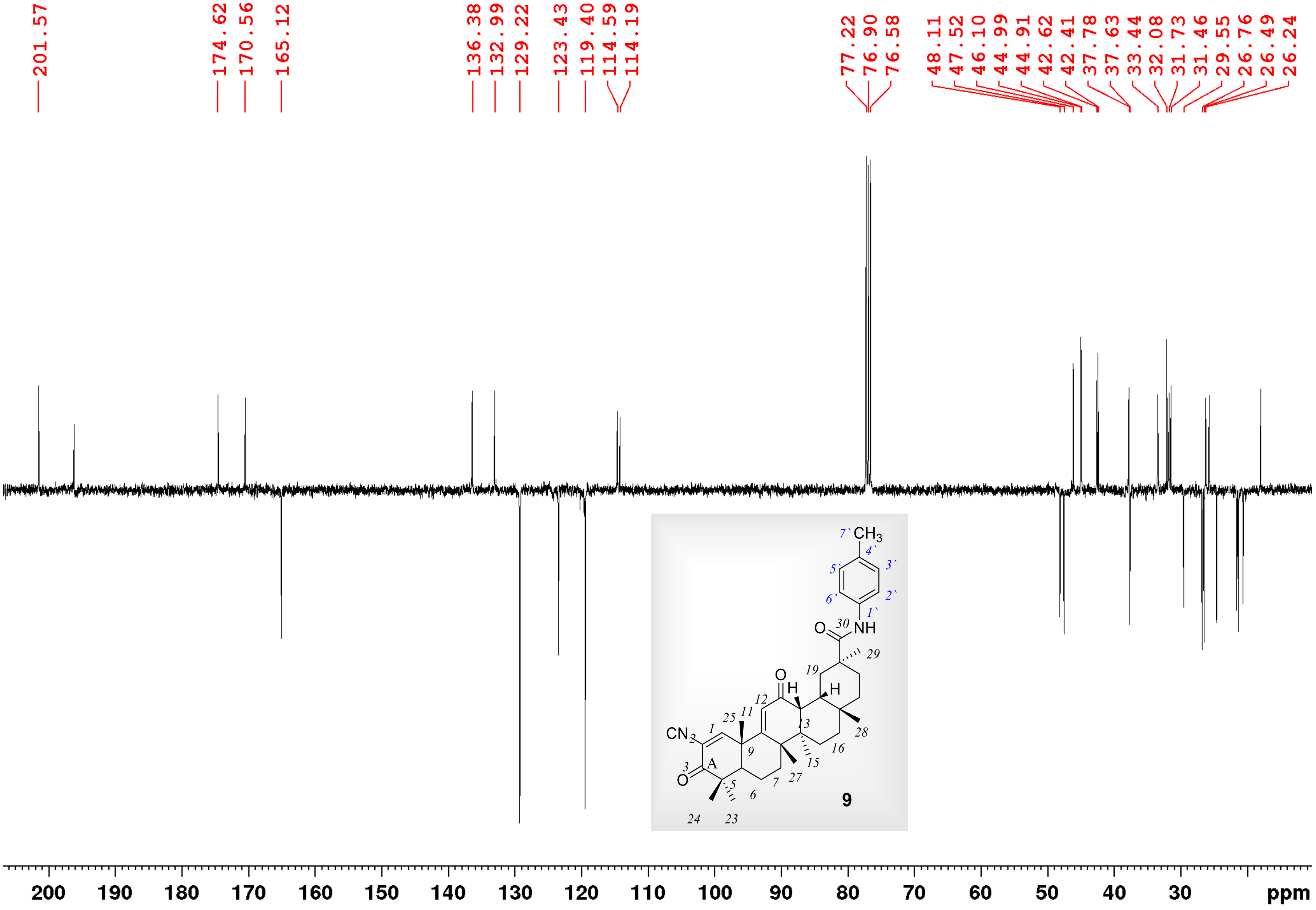

Spectrum of Compound **10**, ^{1}H NMR, 300MHz, $CDCl_3$

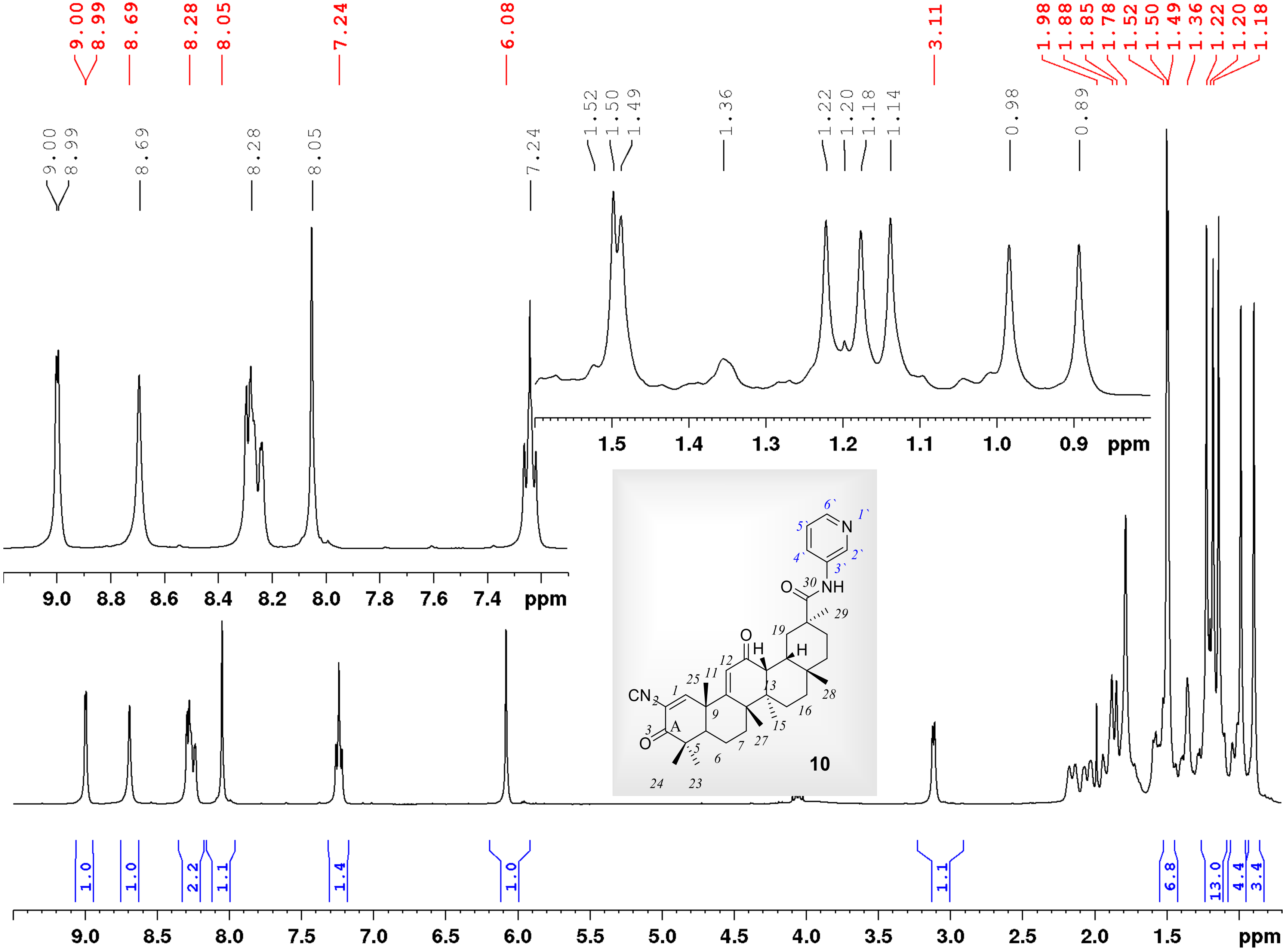

Spectrum of Compound **10,** ^{13}C NMR, BB, 75 MHz, $CDCl_3$

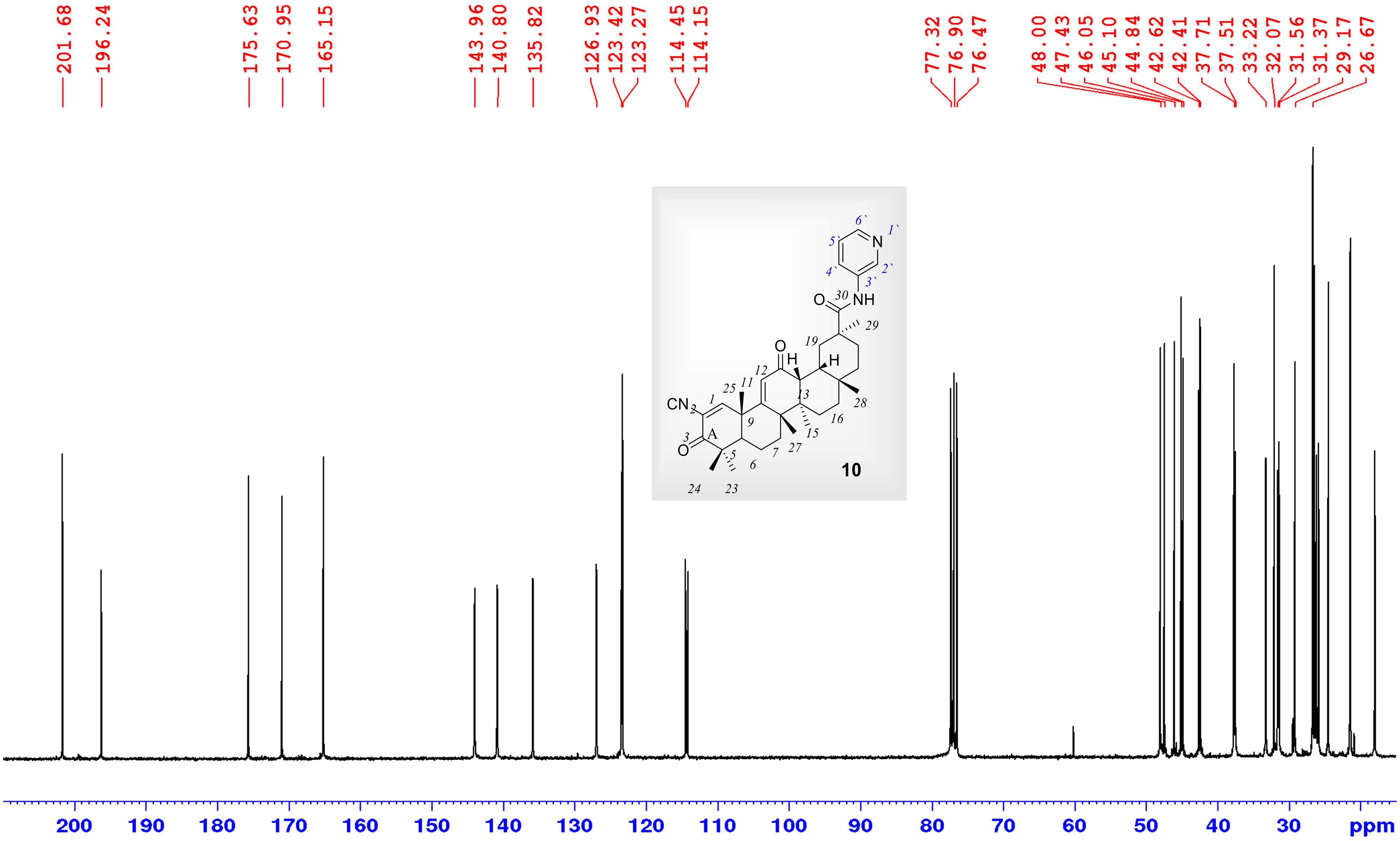

Spectrum of Compound **11,** ^{1}H NMR, 300MHz, $CDCl_3$

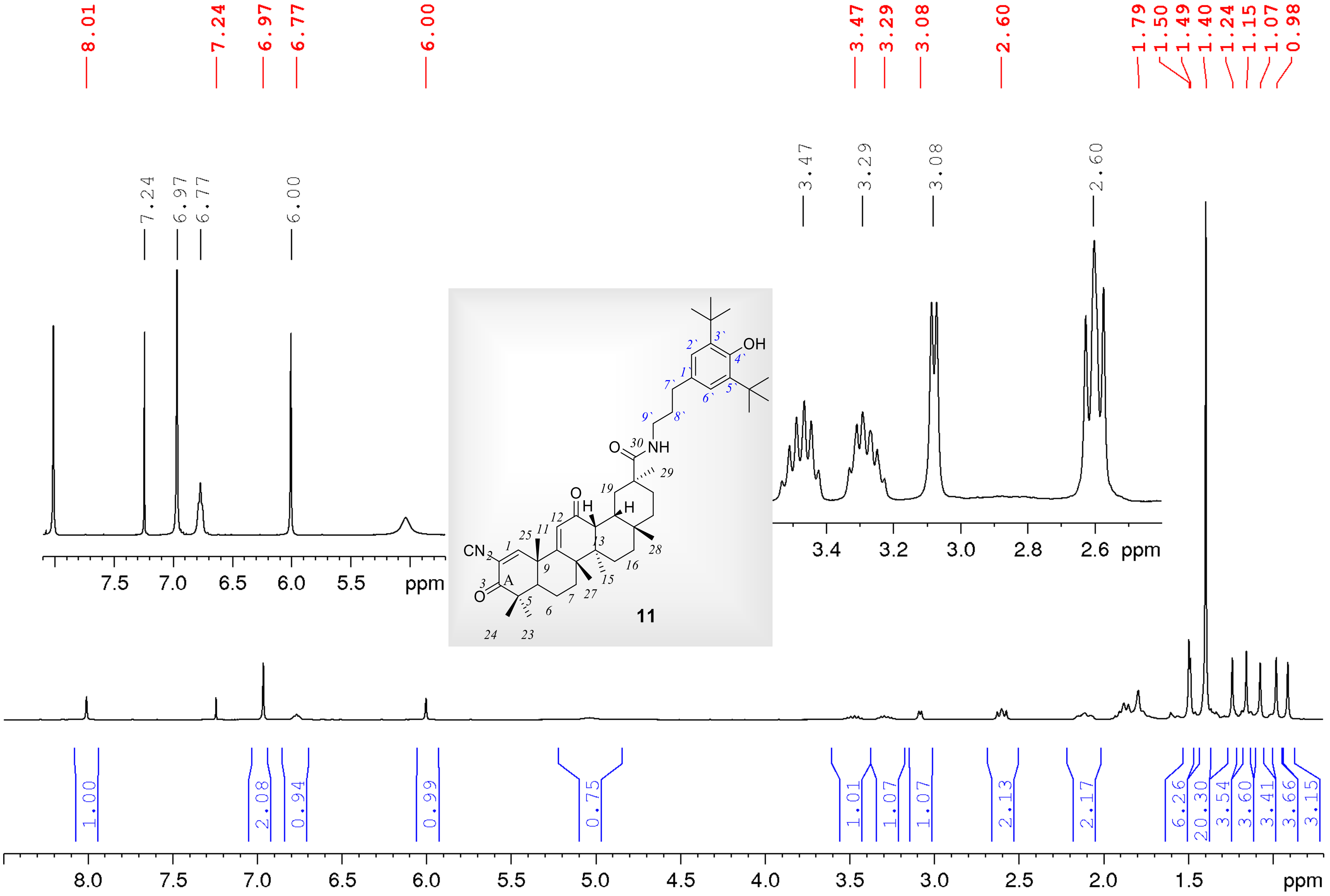

Spectrum of Compound **11,** ^{13}C NMR, JMOD, 75 MHz, $CDCl_3$

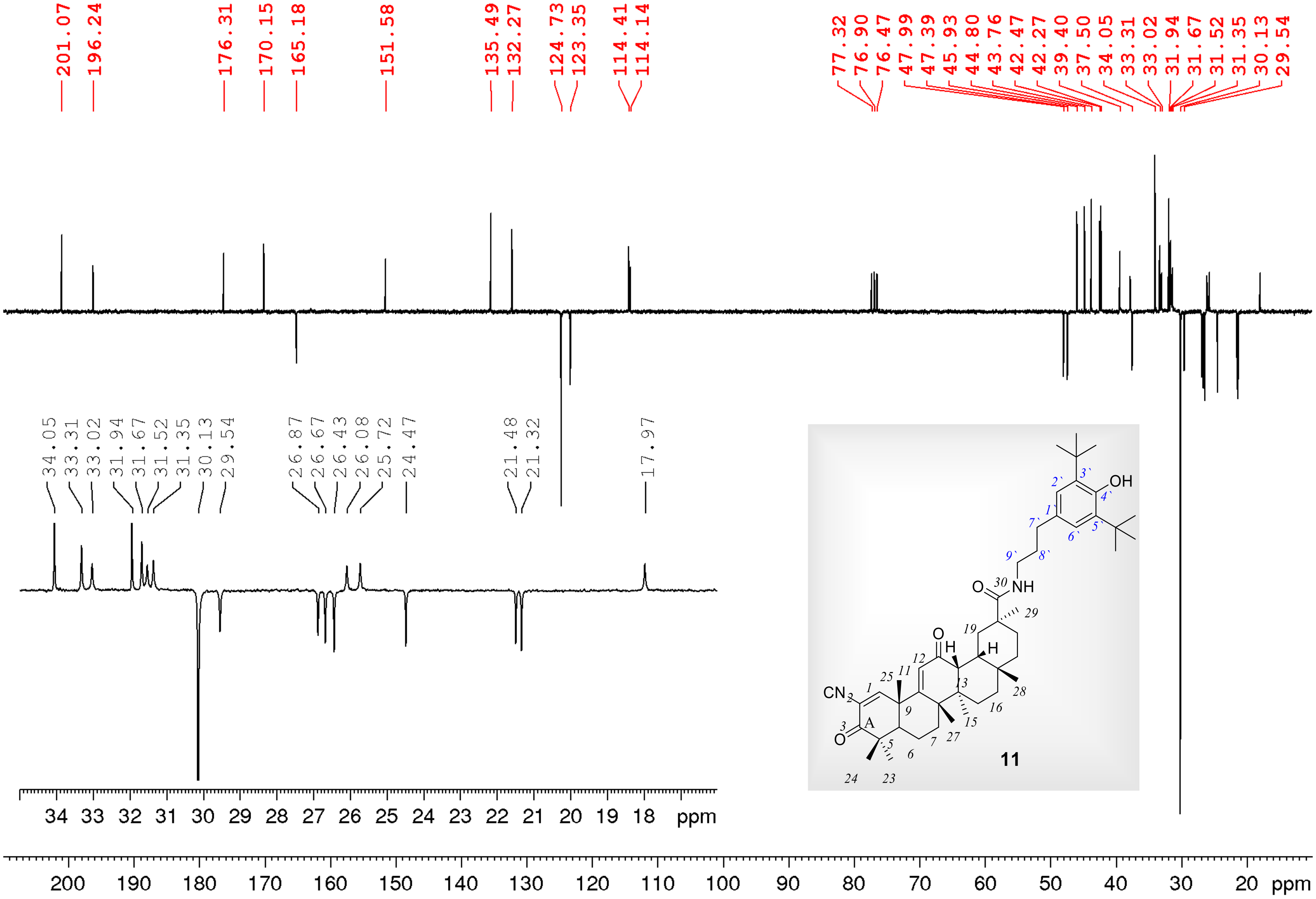
201.07
196.24
176.31
170.15
165.18
151.58
135.49
132.27
124.73
123.35
114.41
114.14
77.32
76.90
76.47
47.99
47.39
45.93
44.80
43.76
42.47
42.27
39.40
37.50
34.05
33.31
33.02
31.94
31.67
31.52
31.35
30.13
29.54
26.87
26.67
26.43
26.08
25.72
24.47
21.48
21.32
17.97
ppm
CN
OH
NH
11

Spectrum of Compound **12,** ^{1}H NMR, 400MHz, $CDCl_3$

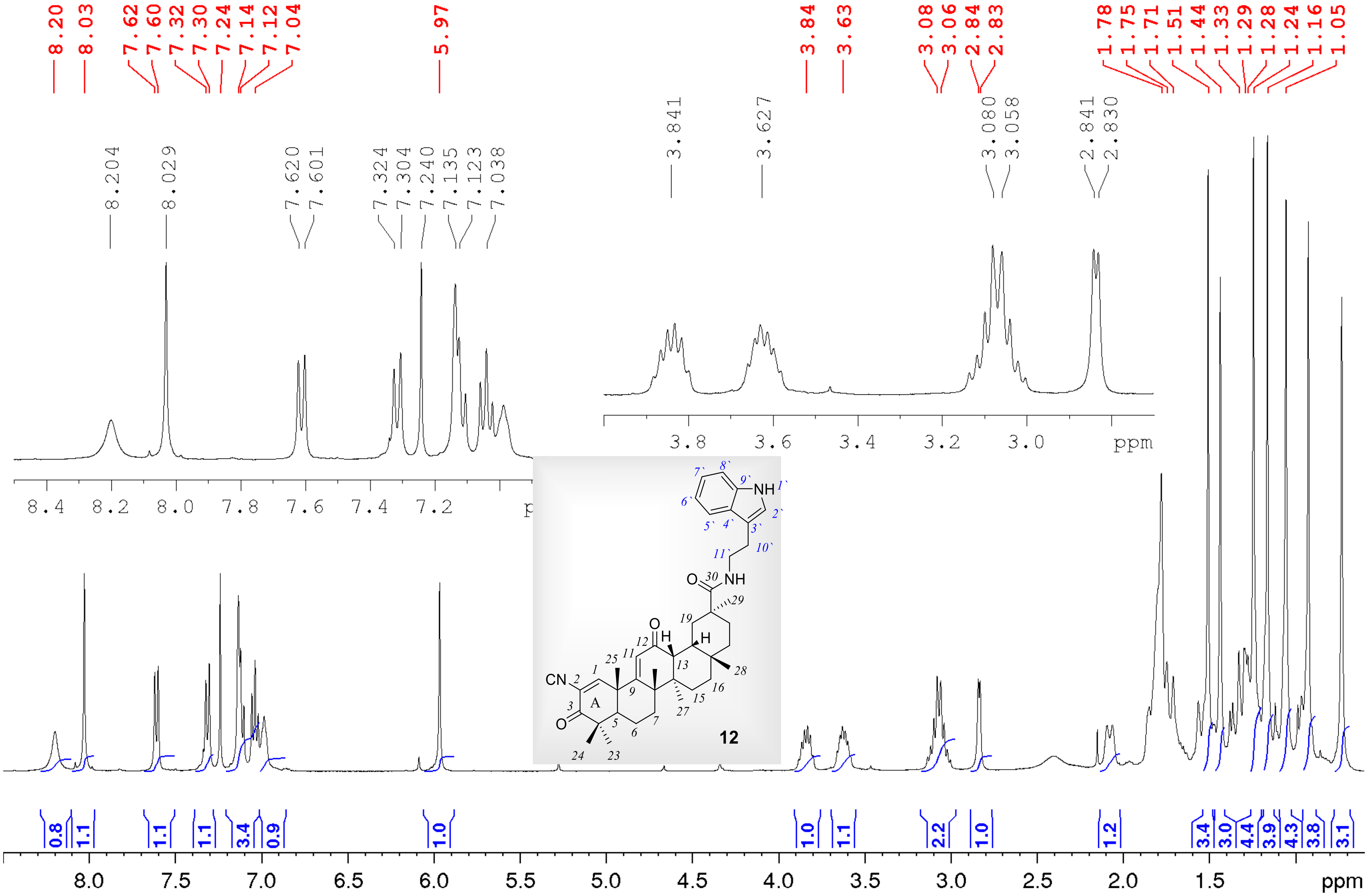

Spectrum of Compound **12,** ^{13}C NMR, JMOD, 400MHz, $CDCl_3$

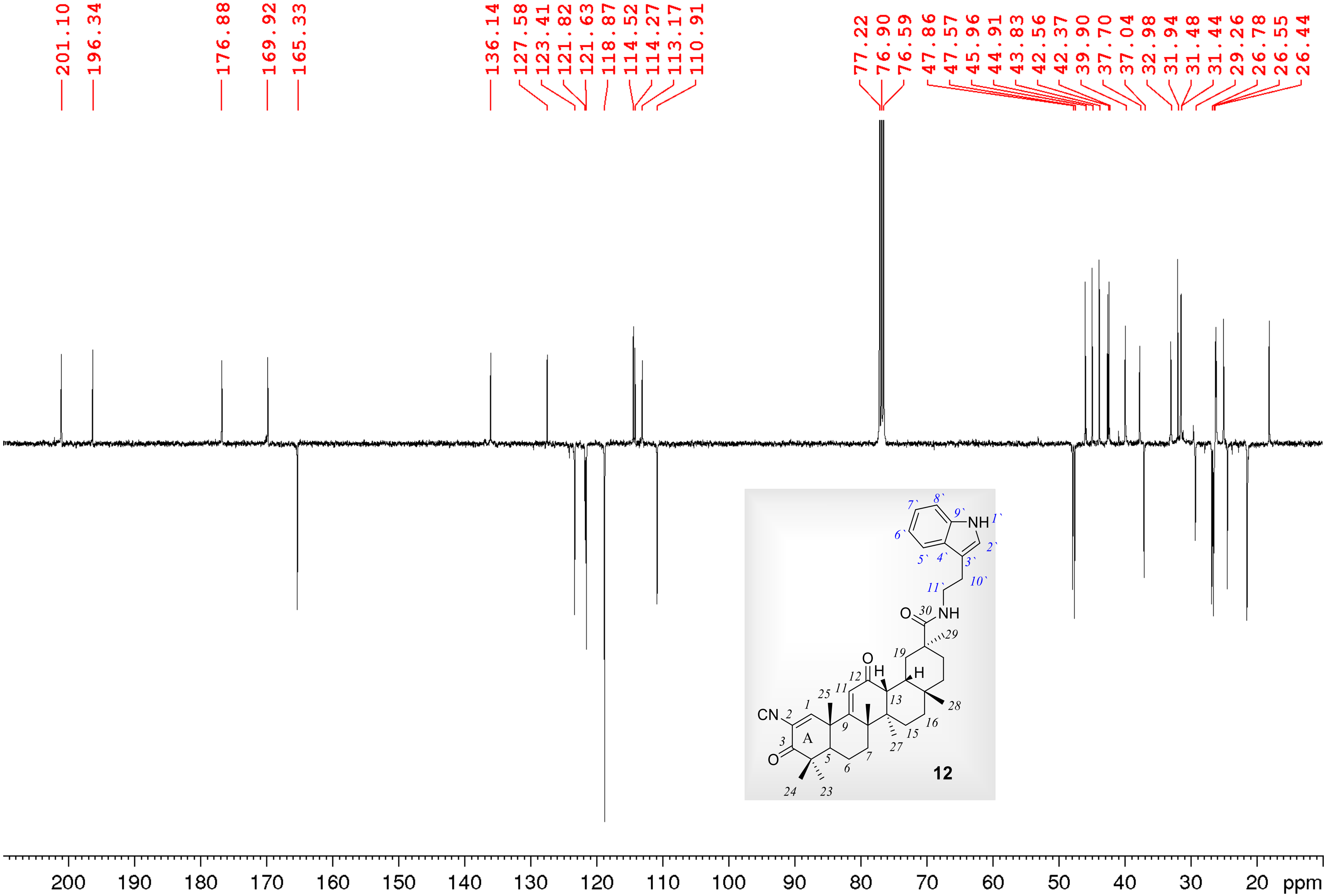
201.10
196.34
176.88
169.92
165.33
136.14
127.58
123.41
121.82
121.63
118.87
114.52
114.27
113.17
110.91
77.22
76.90
76.59
47.86
47.57
45.96
44.91
43.83
42.56
42.37
39.90
37.70
37.04
32.98
31.94
31.48
31.44
29.26
26.78
26.55
26.44
CN
O
NH
12
200 190 180 170 160 150 140 130 120 110 100 90 80 70 60 50 40 30 20 ppm

Supplementary Table S1

Detection parameters of **12** and 2,5-BDPO (internal standard) in MRM mode.

Analyte and its parent ion (Q1 m/z, Da)	Fragment ion (Q3 m/z, Da)	DP, V	CE, V	CXP, V
**12** (634.5)	446.4 [a)]	16	55	10
	144.1 [b)]	36	41	28
	428.5 [b)]	91	51	6
2.5-BDPO (365.3)	176.2 [a)]	146	43	18
	336.2 [b)]	146	31	18
	322.2 [b)]	146	35	16

[a)] Quantifier.

[b)] Qualifier.
